# Associations Between Fathers’ and Mothers’ Psychopathology Symptoms, Parental Emotion Socialization, and Preschoolers’ Social-Emotional Development

**DOI:** 10.1007/s10826-016-0490-x

**Published:** 2016-07-20

**Authors:** Lotte D. van der Pol, Marleen G. Groeneveld, Joyce J. Endendijk, Sheila R. van Berkel, Elizabeth T. Hallers-Haalboom, Marian J. Bakermans-Kranenburg, Judi Mesman

**Affiliations:** Centre for Child and Family Studies, Leiden University, P.O. Box 9555, 2300 RB Leiden, The Netherlands

**Keywords:** Parental psychopathology symptoms, Emotion socialization, Fathers, Mothers, Child social-emotional development

## Abstract

In this study we tested whether the relation between fathers’ and mothers’ psychopathology symptoms and child social-emotional development was mediated by parents’ use of emotion talk about negative emotions in a sample of 241 two-parent families. Parents’ internalizing and externalizing problems were measured with the Adult Self Report and parental emotion talk was observed while they discussed a picture book with their children (child age: 3 years). Children’s parent-reported internalizing and externalizing problems and observed prosocial behaviors were assessed at the age of 3 years and again 12 months later. We found that mothers’ use of emotion talk partially mediated the positive association between fathers’ internalizing problems and child internalizing problems. Fathers’ internalizing problems predicted more elaborative mother–child discussions about negative emotions, which in turn predicted more internalizing problems in children a year later. Mothers’ externalizing problems directly predicted more internalizing and externalizing problems in children. These findings emphasize the importance of examining the consequences of parental psychological difficulties for child development from a family-wide perspective.

## Introduction

Children who grow up in families characterized by parental psychological difficulties are at increased risk for developing social-emotional behavior problems, even when these difficulties are in the subclinical range (Cummings et al. 2005; Papp et al. 2004; Weitzman et al. 2011, see for meta-analytic evidence Connell and Goodman 2002). One of the key mechanisms through which parental symptomatology affects child social-emotional development is maladaptive parenting (Goodman and Godlib 1999). Given that psychological problems often reflect disturbances in emotional functioning (Kring and Bachoroswki 1999), one area of parenting that might be particularly prone to the impact of parental psychological problems is emotion socialization, i.e., parents’ emotional expressiveness, their reactions to child emotions, and parental emotion talk (Eisenberg et al. 1998). Indeed, studies have found that parents with psychological difficulties show less optimal emotion socialization practices such as low sensitive responsiveness to negative child emotions (e.g., Dix et al. 2004). Parental emotion socialization, in turn, plays a central role in several domains of child social-emotional development (Eisenberg et al. 1998). However, the indirect effect of parental psychopathology symptoms on child social-emotional development via parents’ emotion socialization behaviors has rarely been studied. Moreover, parents’ psychological difficulties may not only impair their own emotion socialization behaviors. Theory and research suggest that psychopathology symptoms in one of the parents also influence the other parent’s parenting (e.g., Ponnet et al. 2013). However, to date most studies fail to assume a whole-family perspective, including both parents’ psychological wellbeing as well as their parenting styles.

A large body of research has demonstrated the (prospective) link between parental psychological problems and impaired child social-emotional development (Connell and Goodman 2002; Goodman et al. 2011; Kane and Garber 2004). Although historically most studies on this topic focused on clinical samples (families in which a parent is diagnosed with a psychological disorder), there is increasing evidence that parental psychopathology symptoms at a subclinical level can also have detrimental effects on children’s social-emotional development (Connell and Goodman 2002). For example, various parental psychopathology symptoms such as depressed mood, anxiety, and antisocial traits have been related to children’s internalizing problems such as withdrawn behavior and externalizing problems such as aggression (Breaux et al. 2013; Cummings et al. 2005; Papp et al. 2005). Furthermore, parental psychopathology symptoms have been associated with impaired social skills of children including social withdrawal and a lack of prosocial behavior (Cummings et al. 2005; Elgar et al. 2007). From the perspective of developmental psychopathology it has been proposed that in addition to biological mechanisms (e.g., genetic inheritance; Tsuang and Faraone 1990) and stressful contextual factors (e.g., marital conflict; Cummings et al. 2005; Papp et al. 2004), parental psychological problems affect child development via impaired parenting (Goodman and Godlib 1999). Consistent with this hypothesis, there is ample evidence that depressive symptoms, both at a clinical and subclinical level, in fathers and mothers are associated with various maladaptive parenting behaviors such as coercive parenting, overprotectiveness, and low synchrony during parent–child interaction (Lovejoy et al. 2000; McCabe 2014; Wilson and Durbin 2010). In addition, several studies have shown that other parental psychological disorders such as schizophrenia and anxiety disorders are related to dysfunctional parenting practices, including a lack of parental monitoring and harsh parenting, although it should be noted that most of these studies focused only on mothers (Berg-Nielsen et al. 2002).

Many psychological problems in adults reflect disturbances in emotion processing and emotion expression (Kring and Bachoroswki 1999). For instance, disorders like depression and schizophrenia are marked by a flattened affect (Levin et al. 1985), while anxiety disorders are characterized by the intense experience of negative emotions (Mennin et al. 2002). Further, symptoms of antisocial personality disorder have been related to higher levels of impulsivity and feelings of aggression (Fossati et al. 2002). What these different psychopathology symptoms have in common is that they reflect an increased difficulty with regulating one’s emotions in such a way that they are not overwhelming and potentially harmful to interpersonal relationships (Koole 2009). Given the close link between psychopathology symptoms and emotional functioning, parents’ psychological difficulties may particularly impair their emotion socialization behaviors, i.e., parents’ emotional expressiveness in the presence of their children, parents’ responses to child emotions, and parent–child discussions of emotions (Eisenberg et al. 1998). In line with this idea, Dix’ affective model of parenting states that parents’ emotions are at the heart of both adaptive and maladaptive emotion-related parenting practices with positive and empathic emotions promoting parental warmth, patience, and responsiveness to child emotions, while negative emotions like anger and frustration are thought to lead to parental inattention, avoidance, and hostility (Dix 1991). Relatedly, the developmental psychopathology perspective proposes that parents who experience negative emotions like anxiety and sadness expose their children to the maladaptive thoughts (e.g., ‘I am helpless’) and behaviors (e.g., panic) that go together with these feelings (Goodman and Godlib 1999). It follows from both perspectives that impaired parental emotion socialization behaviors can negatively affect children’s social-emotional development through various processes including modelling negative expressivity, channeling specific emotional responses, emotional insecurity in the home, inadequate scaffolding of child emotion understanding, and shaping children’s schema’s of emotions (Cummings et al. 2014; Eisenberg et al. 1998; Goodman and Godlib 1999). In sum, it is plausible that parental emotion socialization acts as a mediator in the relation between parental psychopathology symptoms and child outcomes.

There is ample evidence for the direct paths that form the basis for this potential mediation effect, namely (1) the path from parents’ psychopathology symptoms to impaired parental emotion socialization behaviors and (2) the path from impaired emotion socialization to maladaptive child social-emotional development. Regarding the first path, several studies have found that parents, mostly mothers, with symptoms of psychopathology express more negative emotions such as distress, contempt, and hostility in the home (e.g., Cummings et al. 2013). Depressed parents in particular have been found to show less affection during parent–child interaction and to be less emotionally involved with their child (Lovejoy et al. 2000; Wilson and Durbin 2010). In a related vein, mothers with psychological difficulties are found to be less sensitive to their child’s emotions (Dix et al. 2004; Nicol-Harper et al. 2007), and more likely to respond in a dismissive manner to their child’s negative feelings (e.g., ignoring, belittling) (e.g., Silk et al. 2011). To date, research on the relation between psychopathology symptoms and parental emotion talk is scarce. There is some observational evidence that mothers with psychopathology symptoms have fewer affective elements in their speech (e.g., encouragement and reassurance) during interaction with their infants than mothers without psychopathology symptoms (Herrera et al. 2004). In contrast, an observation study including mothers and their primary-school-aged children revealed that mothers with psychopathology symptoms were more likely to dwell on negative feelings and to repeatedly discuss stressful experiences with their children (i.e., co-rumination) (Grimbos et al. 2013). It could be that mothers with psychological difficulties focus more on negative emotions during parent–child interaction from toddlerhood onward, when children start talking about emotions themselves.

The second path representing the influence of parents’ emotion socialization behaviors on various domains of child social-emotional development has also been well-documented, albeit again mostly for mothers (e.g., Eisenberg et al. 2003; Grimbos et al. 2013) and to a lesser extent for fathers (e.g., Denham et al. 2010). For example, mothers’ positive emotional expressivity is one of the most robust predictors of adequate social-emotional functioning in children, including adaptive self-regulation and high social competence (Eisenberg et al. 1998). Regarding parents’ direct responses to their child’s emotions, research has shown that mothers who respond in a sensitive manner to negative child emotions like anxiety, for example by comforting the child, directly foster an optimal level of arousal in their children as evidenced by a decrease in heart rate and smooth return to positive affect (Conradt and Ablow 2010; Haley and Stansbury 2003). Further, maternal sensitive responsiveness to child distress during infancy has been found to predict better self-regulation skills in toddlers and preschoolers in challenging situations (e.g., Leerkes et al. 2009). Regarding parental emotion talk, research findings are mixed. That is, several studies have shown that parents who frequently talk about feelings stimulate their child’s understanding of emotions as well as their self-regulation skills and empathic concern for others (Eisenberg et al. 1998). In contrast, more recent evidence indicates that mothers’ emphasis on negative emotions like fear and sadness during parent–child discussions can lead to negative child outcomes such as depressed mood and impaired social skills (Denham et al. 1997; Grimbos et al. 2013; Zahn-Waxler 2000). Also, Cox et al. (2010) showed that adolescent girls whose mothers encouraged them to express their negative feelings developed more internalizing difficulties over time. These findings suggest that a high parental focus on negative feelings during parent–child interaction can stimulate, rather than relieve, social-emotional problems in children.

There is also some empirical evidence supporting the mediating role of parental emotion socialization in the relation between parents’ psychopathology symptoms and child social-emotional development. In two studies mothers’ depression was negatively associated with their responsiveness to child emotions (Feng et al. 2007; Silk et al. 2011). In these studies lower maternal responsiveness was related to children’s higher levels of internalizing problems (Silk et al. 2011) and negative affect (Feng et al. 2007). However, neither study formally tested mediation and both focused only on mothers with or without childhood-onset depression. To our knowledge there is only one study that prospectively tested a mediational pathway from both fathers’ and mothers’ psychopathology symptoms to child social-emotional behavior through parental emotion socialization in a community-based sample. In this study Cummings et al. (2013) found that both parents’ depressive symptoms predicted more child internalizing problems over time as a function of parents’ self-reported negative emotional expressiveness. Although these findings suggest that parental emotion socialization indeed mediates the relation between both parents’ psychopathology symptoms and child social-emotional development, studies using observational data of parental emotion socialization are needed because parents’ psychological difficulties may bias their report on their emotional expressiveness in the home. In addition, previous studies suggesting a mediating role of emotion socialization focused on more implicit emotion socialization practices of which parents are not or only partially aware, i.e., parents’ direct responses to child emotions and their own emotional expressiveness, and little is known about more explicit emotion socialization practices such as parental emotion talk.

Parental psychopathology symptoms may not only affect child social-emotional development through impaired emotion socialization of that particular parent. It is also conceivable that psychological difficulties in one of the parents affect emotion-related parenting practices of both parents, which increases the risk for maladaptive child social-emotional development. According to family systems theories individual family members as well as family sub-systems exert a continuous and reciprocal influence on each other’s daily functioning (Cox and Paley 1997). In a related vein, the cross-over hypothesis proposes that a family member’s affective state influences all family interaction patterns due to the emotional interdependence between family members (Larson and Almeida 1999; Ponnet et al. 2013). Although there is indeed increasing evidence that one parent’s psychological problems affect the other parent’s parenting behaviors (e.g., Beestin et al. 2014; Malmberg and Flouri 2011; Ponnet et al. 2013), it remains unclear whether this effect is negative or positive. Some studies have found evidence for a negative impact of fathers’ and mothers’ psychological difficulties on their partners’ supportive parenting characteristics (Goodman 2008; Malmberg and Flouri 2011; Ponnet et al. 2013). In contrast, there are also studies suggesting that parents (mostly fathers) try to compensate for the lower-quality parenting of their psychologically disturbed partners by intensifying their own positive interactions with their child (Beestin et al. 2014; Edhborg et al. 2003). These mixed findings may be due to the different types of samples that were involved. Generally, studies that found a negative effect of one parent’s psychological difficulties on the other parent’s childrearing behaviors focused on parental psychopathology symptoms in a community-based sample, whereas studies finding evidence for compensatory mechanisms often focused on small groups of families in which one of the parents was diagnosed with depression. Perhaps parents feel more inclined to intensify positive interactions with their children when their partners suffer from severe psychological problems due to the unmistakable negative consequences of parental psychopathology for the ill parent’s child-rearing behaviors, notwithstanding the high level of family stress the other parent is likely to encounter.

Despite the fact that both theory and research suggest that psychopathology symptoms in one of the parents affect *both* parents’ emotion-related parenting behaviors, most studies examining the association between parental psychopathology symptoms and emotion socialization focus on a single parent–child dyad per family. The same is true for studies examining the effect of parental emotion socialization on child social-emotional development. In a related vein, although there is increasing evidence that fathers and mothers differ in both the quantity and content of their emotion socialization behavior (Fivush et al. 2000; Van der Pol et al. 2015; Zaman and Fivush 2013), fathers are underrepresented in studies on the determinants and consequences of emotion socialization during early childhood. Consequently, we know little about the possible unique pathways for fathers and mothers from parental psychological problems to child development through emotion-related parenting. Furthermore, to date research on the effects of parental psychological problems on emotion socialization focused mainly on parents’ internalizing symptoms, such as depressed mood and (to a lesser extent) anxiety, while little attention has been given to the potential negative consequences of parents’ externalizing symptoms like outbursts of anger and impulsive behavior. In this study we investigated the links between fathers’ and mothers’ internalizing and externalizing problems, the degree to which they talk about negative emotions while reading a picture book with their preschoolers, and child internalizing and externalizing problems and prosocial behaviors a year later. Based on the literature, we test three hypotheses. First, because we examined a community-based sample we expected that fathers’ and mothers’ internalizing and externalizing problems would be positively related to their own as well as their partners’ use of emotion talk about negative emotions with their preschoolers. Second, we expected that parent–child discussions of negative emotions would be positively related to child internalizing and externalizing problems, and negatively related to child prosocial behavior. Third, we expected that fathers’ and mothers’ use of emotion talk would mediate the relation between either parent’s psychopathology symptoms and child social-emotional functioning.

## Method

### Participants

This study is part of the longitudinal research project Boys will be boys? which examines the influence of gender-differentiated socialization on the social-emotional development of girls and boys in the first years of life. The current paper focuses on the associations between fathers’ and mothers’ psychopathology symptoms, the degree to which they talk about negative emotions during parent–child discussion of a picture book, and the social-emotional development of preschoolers (51 % boys). This paper reports on data from the third wave, when the children were on average 3.1 years old (*SD* = 0.05), and the fourth wave, when the children were on average 4.0 years old (*SD* = 0.11), which will be referred to as the 3-year wave and the 4-year wave respectively. All children were the second-born child in the family.

Families with two children in the Western region of the Netherlands were selected from municipality records. Families were eligible for participation if the second-born child was around 12 months of age at the time of recruitment and the oldest child was around 2 years older. Exclusion criteria were single parenthood, severe physical or intellectual impairments of parent or child, and having been born outside the Netherlands and/or not speaking the Dutch language. Between April 2010 and May 2011 eligible families were invited by mail to participate in the study. Both parents were asked to participate in one home visit each per year for a period of 4 years. In addition to the home observations, participation in the study included computer testing and filling in questionnaires. Of the 1249 eligible families 31 % (*n* = 390) agreed to participate. The participating families did not differ from the non-participating families on age of fathers (*p* = .13) or mothers (*p* = .83), the educational level of fathers (*p* = .10) or mothers (*p* = .17), and the degree of urbanization of the place of residence (*p* = .77).

At the time of the 4-year wave, 18 families dropped out due to emigration, family issues, or because families considered participation as too demanding. For the current analyses families were excluded when one or both of the parents had missing data on one or both of the pertinent scales for self-reported parental psychopathology symptoms (*n* = 104), or when they did not read the entire emotion picture book with their children (*n* = 2). Further, for each wave families in which both parents had missing data on one or both of the pertinent scales for parent-reported child problem behavior were excluded (*n* = 20), as well as families of which no observational data was available on child prosocial behavior (*n* = 5). If (complete) data on child behavior was available from one of the parents (child problem behavior) or from one of the two home visits (child prosocial behavior), this was taken as the best estimate of the missing scores per wave. Our main findings were similar when these families were excluded from the analyses.

The final sample consisted of 241 families. The participating families did not differ from the excluded families regarding age of mothers at the 4-year wave, degree of urbanization of residence, and fathers’ and mothers’ educational level (all *p*’s > .05). However, fathers in the participating families were slightly older than fathers in the excluded families at both waves (*p*’s < .01) and mothers were slightly older than mothers in the excluded families at the 3-year wave (*p* < .05).

At the 3-year wave, fathers were between 28 and 65 years old (*M* = 39.3, *SD* = 5.4) and mothers were aged between 27 and 48 years (*M* = 36.3, *SD* = 3.9). Most of the parents had finished academic or higher educational schooling (fathers: 77 %, mothers: 81 %). At each wave, most of the participating parents were married or had a registered partnership or cohabitation agreement (>90 %). At the time of the 4-year wave a total of five couples were divorced.

### Procedure

At both waves, each family was visited twice within about 2 weeks, once with the father and the children and once with the mother and the children. The order of father and mother visits was counterbalanced. The participating families received a yearly gift of 30 Euros and small presents for the children. Before each home visit, both parents were asked to individually complete some questionnaires. If parents had not completed the questionnaires at the second home visit, they were sent up to two reminders within 4 weeks after this visit. During the home visit parent–child interactions and sibling interactions were filmed. All visits were conducted by pairs of trained students. Informed consent was obtained from all families. Ethical approval for this research was provided by the Research Ethics Committee of the Institute of Education and Child Studies of Leiden University.

### Measures

#### Parental Psychopathology Symptoms

At the 3-year wave, the scales for Internalizing Problems and Externalizing Problems from the Adult Self Report (ASR: Achenbach and Rescorla 2003) were used to measure parental psychopathology symptoms. Fathers and mothers were asked to fill in on a 3-point scale whether they considered any of the 74 items on the internalizing and externalizing scale (e.g., ‘I cry a lot’, ‘I am mean to others’) to be typical of themselves during the last 6 months. The construct and criterion-related validity as well as the external validity of the ASR have been reported elsewhere (Achenbach and Rescorla 2003). Test–retest reliability of this instrument is good and cross-informant agreement is moderate to high. In this study, the internal consistencies (Cronbach’s Alpha) of the internalizing scale (39 items) were .88 for fathers and .90 for mothers. The internal consistencies of the externalizing scale (35 items) were .81 for fathers and .79 for mothers.

#### Emotion Talk

At the 3-year wave, fathers’ and mothers’ use of emotion talk was measured with a newly developed emotion picture book. This book consists of eight pictures without text or storyline, with drawings of children showing the following facial emotion expressions: anger, fear, sadness, and happiness. In the current study we focused on the emotions anger, fear, and sadness. Each emotion was shown twice; once within a context indicating the cause of the emotion (e.g., deep water causing fear and a broken toy causing sadness) and once displaying only the face of the child. The children on the pictures were drawn in such a way that they were gender neutral (i.e., ambiguous gender, half-long hair). Two versions of the emotion picture book were developed because the children would read the book twice (once with father, once with mother, in counterbalanced order). The two book versions included drawings of the same children but with different hair colors and clothes, and comparable context pictures (e.g., a broken swing or a broken scooter causing sadness). To examine whether the emotions in the emotion picture book were interpreted as they were intended, we asked 67 respondents (36 % male) between 20 and 63 years of age (*M* = 34.0, *SD* = 12.9) with a similar socioeconomic background as the participants in the main study to label the emotions of the children in the pictures. The depicted emotions were labeled correctly in the vast majority of the cases (79–97 %, mean: 92 %). In one of our previous studies with the same sample, meaningful associations were found between parental emotion talk measured with the Emotion Picture Book and child age and parent gender (Van der Pol et al. 2015).

During the home-visits, fathers and mothers were asked to discuss the pictures in the emotion picture book with their child without further directives. Five minutes were allotted for this discussion, but the task could be ended earlier if the parent had finished the book. A coding system was developed for coding parents’ emotion talk, focusing on three aspects of emotion talk: (1) *talking about emotion*, referring to parental comments about the emotions shown in the pictures. (2) *Talking about emotion behavior*, indicating parental statements about the behavioral expression of emotions. (3) *Talking about the cause* of the emotion, referring to comments about contextual factors that can cause an emotion. For each of these three aspects we coded the presence (score 1) versus absence (score 0) of the following types of comments per picture: asking questions, labeling, referring to the child’s experiences, referring to others’ experiences (see Table 1 for examples). The potential score range of the total score for use of emotion talk was 0–12 with a score of 12 referring to the presence of each of the four types of emotion talk for each of the three aspects of emotion talk.

**Table 1:** Examples of emotion talk

Variable of interest	Example
Emotion talk	
Talking about emotion	
Asking	“How does she feel?”
Labeling	“This child is angry”
Involving child	“Yesterday, you got angry, too”
Involving other	“Your sister is sometimes sad”
Talking about emotion behavior	
Asking	“Is he crying?”
Labeling	“She’s stamping her feet”
Involving child	“You were also crying the other day”
Involving other	“He’s screaming, just like John”
Talking about the cause	
Asking	“Why is he screaming?”
Labeling	“Her swing is broken, that’s why she’s so sad”
Involving child	“Are you afraid of the deep water?”
Involving other	“Lisa gets angry too when she isn’t allowed to eat candy”

A group of 16 undergraduate students rated the 482 videos (two dyads in 241 families) on parental emotion talk. After being trained on a set of 26 videos, each student completed a reliability set of 30 videos. Interobserver reliability based on this reliability set was adequate with intraclass correlations (single rater, absolute agreement) for all pairs of coders being higher than .70. Fathers and mothers within the same family were coded by different coders to guarantee independency among ratings. No coder rated a parent twice across the two waves.

#### Child Behavior Problems

At both waves, the scales for Internalizing Behavior and for Externalizing Behavior from the Child Behavior Checklist for preschoolers (CBCL/1½–5; Achenbach and Rescorla 2000) were used to measure behavior problems of the child. Both fathers and mothers were asked to indicate whether they had observed any of the described 55 behaviors on the internalizing and externalizing scale in the last 2 months on a 3-point scale (0 = *not true*, 1 = *somewhat or sometimes* *true*, 2 = *very true or often true*). Previous research has demonstrated the construct and criterion-related validity as well as the external validity of this instrument (Rescorla 2005). Also, the CBCL has proven to have good test–retest reliability and adequate cross-informant agreement. In this study, the internal consistencies (Cronbach’s Alpha) ranged from .76 to .80 for the internalizing scale (19 items) and from .90 to .93 for the externalizing scale (36 items). At every wave, the CBCL scores of fathers and mothers were significantly correlated for each scale (.42–.44, *p*s < .01). To obtain a composite measure for children’s behavior problems, father and mother scores were averaged for each scale at each wave.

#### Child Prosocial Behavior

At both waves, sharing was used as a measure of child prosocial behavior. Children’s tendency to share toys, stickers, or treats with peers, siblings, and parents has been observed extensively in previous research as an indicator of prosocial behavior (e.g., Leimgruber et al. 2012; Lipps Birch and Billman 1986; Schmidt and Sommerville 2011), and it has been linked to children’s perspective-taking skills (Eisenberg and Miller 1987). In the current study, the children received a small box of raisins (a common children’s treat in the Netherlands) and were requested by the experimenter to share these with their older sibling. If one of the children did not like raisins, an alternative treat was given after consulting the parent (mostly small pieces of ginger bread). The sharing task was administered during both the father and mother visits. During the first minute of the task, the parent was present, but was instructed not to intervene. After this minute, the parent was free to intervene if he or she considered this necessary. Here we focus on child prosocial behavior during the whole task. Using child prosocial behavior during the first minute of the task, based on a smaller sample in which neither parent interfered in the first minute at both waves (*n* = 124), yielded comparable results as using child prosocial behavior throughout the task.

The task was filmed and the numbers of treats eaten by the child and shared with his or her older sibling were counted. Treats shared with or by the parent were not counted; when a child took treats back from the older sibling, these were subtracted from the total number of shared treats. Two groups of 15 undergraduate students in total (six at the 3-year wave and nine at the 4-year wave) rated the 964 videos (two home visits at two waves for 241 families) on sharing. After being trained on a set of 16 videos, each student in each group completed a reliability set (n = 30) with 50 % overlap between the two sets (coders who rated the 4-year wave completed a reliability set including 15 videos of that particular wave and 15 videos that were also in the reliability set of the coders who rated the 3-year wave). Interobserver reliability based on the reliability set was adequate; the intraclass correlations (single rater, absolute agreement) between all pairs of coders were equal to or above .70. Children’s sharing during the father and the mother home visit were coded by different coders to guarantee independency among ratings. No coder rated a child twice either within or across waves. In addition, sharing was rated by a group of coders who did not code parental emotion talk of either parent in either wave.

From the total number of treats that were eaten by the two children, we calculated the proportion of treats given to the older sibling. Sharing behavior was significantly correlated between the visit with the father and the visit with the mother at the 3-year wave (*r* = .29, *p* < .01) as well as the 4-year wave (*r* = .22, *p* < .01). We therefore used a mean score for children’s sharing behavior at each wave.

### Data Analyses

All measures were inspected for possible outliers that were defined as values more than 3.29 *SD* above or below the mean (Tabachnick and Fidell 2012). Outliers were found for fathers’ externalizing problems (*n* = 3), mothers’ externalizing problems (*n* = 2), maternal emotion talk (*n* = 2), child internalizing problems at the 3-year wave (*n* = 2) and the 4-year wave (*n* = 1), and child prosocial behavior at the 4-year wave (*n* = 1). The outlying values were winsorized, meaning that they were given a score that was no more extreme than the most extreme value that fell within the accepted range of a normal distribution. Because both parents’ internalizing problems were positively skewed, logarithmic (log10) transformations of scores were used to approach normal distributions (Tabachnick and Fidell 2012).

Pearson correlation coefficients were computed to examine the associations between all variables. To examine whether fathers’ and mothers’ internalizing and externalizing problems had an indirect effect on child problems through parents’ use of emotion talk about negative emotions, a set of mediation analyses was performed. The Preacher and Hayes approach to test mediation was applied using the macro package for SPSS available online which allows for multiple predictors and mediators (Hayes 2013). This method adopts the bootstrapping approach that does not assume that the sampling distributions of the indirect effect are normal, unlike the Sobel test (Preacher and Hayes 2004). Sampling distributions are estimated from random samples based on the original data. Five thousand bootstrap resamples were taken and 95 % BC confidence intervals were computed (Preacher and Hayes 2008). In total, three regression analyses were performed to test the mediation hypothesis with, respectively, children’s externalizing problems, internalizing problems, and prosocial behavior as outcomes at the 4-year wave. Each regression analysis included the pertinent child characteristic (as control variable) and fathers’ and mothers’ internalizing and externalizing problems at the 3-year wave as predictors, and fathers’ and mothers’ emotion talk at the 3-year wave as mediators. Thus, in each mediation analysis we examined the direct and indirect effects of each parent’s psychopathology symptoms while controlling for the other parent’s psychopathology symptoms, and the same is true for the direct effects of fathers’ and mothers’ emotion talk.

## Results

The means, standard deviations, and bivariate correlations are presented in Table 2. Parental internalizing and externalizing problems were positively correlated for fathers as well as for mothers. In addition, fathers’ and mothers’ psychopathology symptoms were positively correlated and mothers reported more internalizing problems than fathers, *t*(240) = −2.16, *p* < .05, *d* = −0.17. No mean differences between fathers’ and mothers’ externalizing problems were found. Fathers’ and mothers’ emotion talk were positively correlated and their mean scores did not significantly differ from each other. Further, fathers’ internalizing problems were positively related to maternal emotion talk. Children’s behavior problems were highly correlated between waves and their scores on the internalizing and externalizing problem scales were positively associated both within and across waves. In addition, children’s prosocial behaviors at the 3-year wave were positively related to their internalizing problems at the 4-year wave. Fathers’ and mothers’ psychopathology symptoms were positively associated with children’s internalizing and externalizing problems. Finally, maternal emotion talk was positively associated with child internalizing problems at the 4-year wave.

**Table 2:** Summary of means, standard deviations, and correlations for all study variables (n = 241)

	1	2	3	4	5	6	7	8	9	10	11	*M*	*SD*
3-Year wave													
1. Father INT												9.11	7.21
2. Father EXT	.52**											7.55	4.99
3. Father EM	.04	.09										2.50	0.73
4. Mother INT	.24**	.16*	.10									10.44	8.31
5. Mother EXT	.28**	.16*	.11	.63**								7.34	4.76
6. Mother EM	.17**	.09	.16*	.04	−.02							2.59	0.74
7. Child INT	.30**	.26**	−.03	.19**	.18**	.08						5.18	3.31
8. Child EXT	.42**	.37**	.08	.30**	.38**	.09	.45**					20.23	8.61
9. Child PRO	.02	−.06	.00	.07	.02	.04	.06	.03				0.50	0.16
4-Year wave													
10. Child INT	.36**	.20**	−.03	.24**	.29**	.15*	.68**	.39**	.14*			4.52	3.22
11. Child EXT	.35**	.30**	.02	.26**	.36**	.07	.38**	.72**	.04	.53**		16.46	8.68
12. Child PRO	−.10	.01	.01	−.03	−.09	.10	.03	.00	.03	−.07	−.05	0.53	0.15

*INT* internalizing problems,* EXT* externalizing problems,* EM* emotion talk,* PRO* prosocial behavior. To facilitate interpretation, the non-transformed scores are presented. Child prosocial behavior is the proportion of treats shared with the older sibling

* *p* < .05; ** *p* < .01

**Fig. 1:**
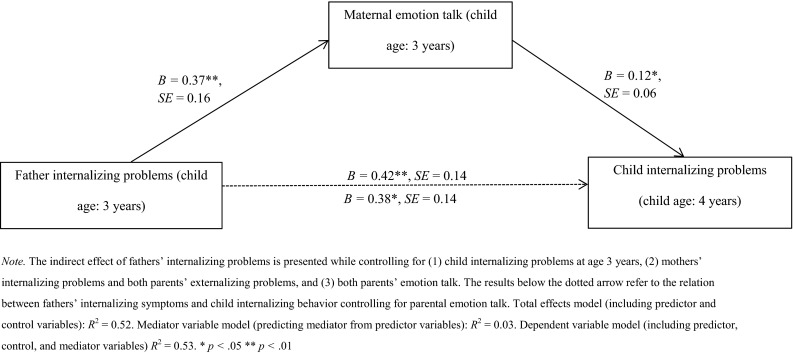
Mediation model predicting child internalizing problems at 4 years of age from fathers’ internalizing problems through maternal emotion talk about negative emotions, both at 3 years of age (*n* = 241)

Using the SPSS macro package (Hayes 2013), we examined whether fathers’ and mothers’ psychopathology symptoms at the 3-year wave had an indirect effect via either parent’s use of emotion talk about negative emotions at the 3-year wave on child internalizing and externalizing problems and prosocial behaviors at the 4-year wave, while controlling for these child behaviors a year earlier. Because the macro package allows for multiple predictors and mediators in a single model, a total of three regression analyses were performed to test the mediation hypothesis for each of the child outcome behaviors (internalizing, externalizing, and prosocial) including fathers’ and mothers’ internalizing and externalizing problems as predictors and fathers’ and mothers’ emotion talk as mediators. In the mediator variable model, which was the same for all three analyses (predicting fathers’ emotion talk and mothers’ emotion talk from each parent’s internalizing problems and externalizing problems, controlling for child behavior at the 3-year wave), fathers’ internalizing problems positively predicted maternal emotion talk about negative emotions (*B* = 0.36–0.38, *SE* = 0.16, *p*s < .05). In the first dependent variable model mothers’ emotion talk predicted more child internalizing problems (*B* = 0.12, SE = 0.06, *p* < .05). Further, the indirect path from fathers’ internalizing problems to child internalizing problems through maternal emotion talk was significant as well (*B* = 0.04, *SE* = 0.03, *BC* 95 % CI = 0.003, 0.14). The direct effect of fathers’ internalizing problems on child internalizing problems remained significant (*B* = 0.38, *SE* = 0.14, *p* < .01). Figure 1 shows the complete mediation model for child internalizing problems. Regarding children’s externalizing problems and prosocial behavior, the indirect paths from fathers’ and mothers’ psychopathology symptoms through parents' use of emotion talk were not significant for either parent. We checked whether fathers’ and mothers’ emotion talk and child social-emotional behaviors varied between boys and girls. Because this was only the case for one out of eight outcome variables, we decided not to conduct separate mediation analyses for boys and girls given the increased risk of a Type 1 error.

In addition to the direct and indirect effects of fathers’ internalizing problems, mothers’ externalizing problems had a direct effect on child internalizing and externalizing problems both with and without controlling for both parents’ emotion talk. That is, more externalizing problems of the mother predicted more internalizing problems (*B* = 0.03, SE = 0.01, *p*s < .05) and externalizing problems of the child (*B* = 0.21–0.22, SE = 0.11, *p*s < .05). We found no effects of either parent’s psychopathology symptoms on child prosocial behavior.

## Discussion

Our study provides insight in the intergenerational transmission of parental psychopathology to child behavior problems via emotion socialization. Mother–child discussions of negative emotions when children were 3 years of age partially mediated the positive relation between fathers’ internalizing psychological problems at age 3 years and child internalizing problems a year later. More internalizing problems of the father predicted more elaborative mother–child conversations about negative emotions, which in turn predicted more child internalizing problems. Further, more externalizing problems of the mother directly predicted more internalizing and externalizing problems in preschoolers.

Contrary to our expectations we found no relation between fathers’ and mothers’ psychopathology symptoms and their own use of emotion talk with their preschoolers. This finding might be due to the fact that most parents in our study were highly educated, which can result in parents’ higher awareness of their own psychological issues and the potential consequences of these issues for their child’s social-emotional development. This awareness may in turn stimulate parents to protect their children from their psychological difficulties, thus preventing a spill-over effect of parents’ psychopathology symptoms to their parenting skills. Indeed, there is evidence that parental educational level acts as an important protective factor in the association between parental psychopathology and maladaptive parenting (Greeff et al. 2006; Serbin et al. 1998).

Although we found no association between parents’ symptoms of psychopathology and their own use of emotion talk, fathers’ internalizing problems did predict more elaborative mother–child conversations about negative emotions. Parents whose partners have psychological problems often experience high levels of family stress (Logan 2011), which increases the risk for maladaptive parent–child interaction patterns such as parent–child co-rumination, which refers to excessively discussing negative feelings, stressful events, and personal issues (Grimbos et al. 2013). In a related vein, consistent with theories on emotional contagion according to which intimate partners are highly vulnerable to each other’s emotions (Goodman and Shippy 2002), it is conceivable that mothers are biased toward negative emotions due to their partners’ psychological difficulties, leading mothers to talk more about these emotions with their children. From the perspective of the compensatory hypothesis (Nelson et al. 2009), it could also be that mothers try to protect their child from the negative consequences of being exposed to the psychological difficulties of their partner by elaborating more on negative emotions during parent–child discussions to increase children’s emotional understanding. It should be noted, however, that because parents’ psychopathology symptoms and emotion talk were measured simultaneously, we cannot rule out the alternative explanation that mothers’ tendency to focus on negative feelings predisposed them to select a partner with emotional difficulties.

The fact that fathers’ use of emotion talk was not related to mothers’ psychopathology symptoms might be due to our focus on the way parents *talk* about emotions with their children rather than the way parents express their emotions and their reactions to child emotions. Although previous studies have shown that mothers’ psychological difficulties influence fathers’ parenting practices in terms of affect expression during parent–child interactions and their sensitive responses to child signals (e.g., Goodman 2008; Ponnet et al. 2013), these studies did not include parent–child discussions of emotions. A large body of research has shown that women talk more about their emotional experiences with others than do men (Brody and Hall 2008). There is also evidence that mothers are more likely than fathers to discuss family-related issues (e.g., marital conflict) with their children (Peris et al. 2008). These findings suggest that a partner’s psychological problems may trigger mothers more than fathers to discuss negative emotions with their children. This is not to say that mothers are more affected by family stressors including the partner’s psychological problems than fathers. In contrast, differences between fathers’ and mothers’ parenting stress when faced with family stressors such as children’s behavior problems or the birth of a preterm infant are often found to be negligible (Deater-Deckard 1998; Schappin et al. 2013; Theule et al. 2012).

Consistent with our expectations, parental psychopathology symptoms in part predict child social-emotional development via parental emotion socialization. Mothers’ use of emotion talk mediated the positive association between fathers’ internalizing problems and children’s internalizing problems with more elaborative mother–child discussions about negative emotions at age 3 years predicting more internalizing problems in their children a year later. At first glance, this finding appears to contradict previous research on parental emotion socialization as well as various theories on supportive parenting, proposing that an open and accepting attitude toward negative child emotions and the willingness to talk about such feelings foster children’s adequate understanding and regulation of emotions, and empathic concern for others (Eisenberg et al. 1998; Gottman et al. 1996; Kochanska 2002; Mesman et al. 2012). However, in line with the perspective of emotional contagion (Goodman and Shippy 2002), mothers’ focus on negative emotions can carry the risk of arousing children’s cogitation on stressful experiences and the accompanying feelings (Zahn-Waxler 2000). Indeed, research on parent–child co-rumination has shown that mothers’ tendency to dwell on negative feelings with their children is positively related to children’s internalizing characteristics such as anxiety and sadness (Calmes and Roberts 2008; Grimbos et al. 2013; Waller and Rose 2010).

In addition to an indirect effect via maternal emotion talk, fathers’ internalizing problems also directly predicted more internalizing problems in their children. Further, mothers’ externalizing problems directly predicted more internalizing and externalizing problems in children. The fact that less optimal child outcomes were related to different types of psychopathology symptoms in fathers and mothers might reflect children’s internalized gender role standards about appropriate behaviors of males and females. In most Western countries, women are expected to express more internalizing emotions like sadness and anxiety than men, whereas men are expected to express more disharmonious emotions (e.g., anger) that assert one’s own interests over others’ (Brody 2000; McIntyre and Pope Edwards 2009). Already from the age of 2 years children start internalizing gender-typed ideas about which behaviors are appropriate for men and women (Poulin-Dubois et al. 2002). This may lead preschoolers to consider fathers’ internalizing problems as less normal and thus more puzzling than mothers’ internalizing problems, while the opposite may be true for externalizing problems. Given that most parents in our study had subclinical levels of psychopathology symptoms, it could be that only symptoms that contradict gender stereotypes had a negative impact on children as these symptoms may cause more confusion and anxiety than symptoms that are in line with gendered ideas about emotion expression in men and women. Consistent with this idea, low levels of mothers’ physical aggression (spanking) have been found to be related to child problem behavior, while for fathers only high levels of physical aggression predicted more child problem behavior (Mackenzie et al. 2013). To clarify whether children’s internalized ideas about which emotions are more accepted in males and females indeed influence the link between parental psychological difficulties and child social-emotional development, research into potentially gendered ways in which children experience and conceive their fathers’ and mothers’ psychopathology symptoms is needed.

The current study has some limitations. First, the direction of the positive association between fathers’ internalizing problems and mothers’ use of emotion talk is equivocal because both parental characteristics were measured at the same time. Cross-lagged longitudinal designs are necessary to gain more insight in the possible bidirectionality of the association between parents’ psychopathology symptoms and their partners’ emotion socialization behavior. Second, the coding of parent–child discussions of negative emotions did not take emotion-related comments of the child into account, which may explain the fact that parents’ mean scores on emotion talk were relatively low. In addition, we did not code the content and affective tone of parental emotion talk, which could have provided further insight in the positive relations we found between fathers’ psychopathology symptoms and maternal emotion talk and between maternal emotion talk and child internalizing problems. Research has shown that differences in level of attention for emotions during parent–child discussions, regardless of content and tone, are associated with various aspects of child social-emotional development (Jenkins et al. 2003; Perez Rivera and Dunsmore 2011). Nevertheless, the degree to which parental emotion talk is related to positive social-emotional functioning in children is likely to be influenced by the quality of the interaction (Eisenberg et al. 1998). Third, parents’ psychological problems and children’s behavior problems were both measured with parent reports. There is accumulating evidence that the way parents perceive and evaluate their child’s behavior is influenced by parents’ own emotional wellbeing (e.g., Chilcoat and Breslau 1997). Although we used aggregate scores based on father-reports and mother-reports of child behavior problems and we observed child prosocial behavior in the home, future studies should include observations of child externalizing problems and interviews of child internalizing problems (e.g., the Berkeley Puppet Interview; Ringoot et al. 2013) to avoid potential response biases based on parents’ own psychological difficulties. Finally, as an indicator of child prosocial behavior we counted the number of treats shared and eaten, and we did not observe any other aspect of the child’s sharing behavior, nor did we take the behavior of the older sibling or parent into account. Given that children were requested by the experimenter to share treats with their siblings and parents were free to intervene after the first minute of the task, it could be that we captured compliance or experimenter-pleasing behavior in children rather than altruism. However, this sharing can still be considered as a form of prosocial behavior in that it is at least partly intended to benefit others (Batson and Powell 2003; Warneken and Tomasello 2007). Despite these limitations this study extends previous research by formally testing a mediation model including both mothers’ and fathers’ observed parenting behavior, and examining the relation between parents’ psychological problems and their own as well as their partner’s emotion socialization behavior.
